# Hybrid-Actuating Macrophage-Based Microrobots for Active Cancer Therapy

**DOI:** 10.1038/srep28717

**Published:** 2016-06-27

**Authors:** Jiwon Han, Jin Zhen, Van Du Nguyen, Gwangjun Go, Youngjin Choi, Seong Young Ko, Jong-Oh Park, Sukho Park

**Affiliations:** 1 School of Mechanical Engineering, Chonnam National University, Gwangju, Korea.

## Abstract

Using macrophage recruitment in tumors, we develop active, transportable, cancer theragnostic macrophage-based microrobots as vector to deliver therapeutic agents to tumor regions. The macrophage-based microrobots contain docetaxel (DTX)-loaded poly-lactic-co-glycolic-acid (PLGA) nanoparticles (NPs) for chemotherapy and Fe_3_O_4_ magnetic NPs (MNPs) for active targeting using an electromagnetic actuation (EMA) system. And, the macrophage-based microrobots are synthesized through the phagocytosis of the drug NPs and MNPs in the macrophages. The anticancer effects of the microrobots on tumor cell lines (CT-26 and 4T1) are evaluated *in vitro* by cytotoxic assay. In addition, the active tumor targeting by the EMA system and macrophage recruitment, and the chemotherapeutic effect of the microrobots are evaluated using three-dimensional (3D) tumor spheroids. The microrobots exhibited clear cytotoxicity toward tumor cells, with a low survivability rate (<50%). The 3D tumor spheroid assay showed that the microrobots demonstrated hybrid actuation through active tumor targeting by the EMA system and infiltration into the tumor spheroid by macrophage recruitment, resulting in tumor cell death caused by the delivered antitumor drug. Thus, the active, transportable, macrophage-based theragnostic microrobots can be considered to be biocompatible vectors for cancer therapy.

Micro/nanoparticle (NP)-based therapy delivers particles containing therapeutic agents to a target area using the enhanced permeability and retention (EPR) effect^1^, which has been developed for various biomedical applications, including tumor treatments^1^. However, this method has some limitations in cancer therapy because it depends only on the leakage property of tumor vessels, which can be influenced by several factors^2^,^3^,^4^,^5^. These factors include heterogeneous blood flow by an abnormal vascular network, permeability due to a structural abnormality of the tumor’s blood vessels, gradient cell proliferations due to heterogeneity of the blood supply, and high intratumoral pressure^1^,^2^,^3^,^4^,^5^. Therefore, the particles themselves cannot penetrate into the avascular central region of a tumor using the EPR effect. Moreover, because the size and surface features of the particles can affect their delivery efficacy into the tumor using the EPR effect, smaller and positively charged particles show greater accumulation in tumors^2^,^3^. Particles larger than 100 nm or with positively charged surfaces can be cleared by phagocytic uptake and hepatic filtration^2^, and particles smaller than 10 nm can also be excreted by the kidney^2^. Thus, the efficacy of drug delivery using particle-based therapy with EPR effects showed an increase of only 20–30% in tumors compared with normal organs^1^.

To overcome these limitations, cancer therapy using immune cells has been proposed, wherein the immune cells function as vectors or carriers for the delivery of therapeutic agents^6^. The immune cells have great potential abilities, such as tumor targeting properties, localization of therapeutic agents to the tumor lesion, and prevention of the spread of therapeutic agents into normal tissues^6^. Among these immune cells, macrophages, which make up a major portion of the innate immune system, perform diverse functions, such as phagocytosis of foreign pathogens, injured cells, and cell debris; activation of the immune system through antigen presentation; and secretion of various cytokines. As such, they are essential for immune responses^7^, including against tumors^8^. In the tumor microenvironment, many peripheral macrophages are infiltrated into or around the tumor mass by its chemoattractive gradient^8^, and these tumor infiltrated macrophages are referred to as tumor-associated macrophages (TAMs)^9^. TAMs can make up over 70% of the cells in breast carcinoma^9^. TAMs can traverse the blood–brain barrier (BBB) and vascular endothelium, penetrate into avascular regions, eventually can exist not only in the vascular region, but also in the avascular regions, such as infarction^10^ and tumor hypoxic regions^8^. In particular, TAMs have been reported to accumulate in high density in the central hypoxic regions of tumor tissues^8^. These TAMs exert cytotoxic effects on tumor cells in the early stages of the immune response^11^, but TAMs were also associated with poor prognoses which they might promote the propagation of malignant solid tumors, including breast cancer, non-small-cell lung cancer, and Hodgkin’s lymphoma^8^,^9^,^10^,^11^,^12^,^13^. It has been reported that these macrophages might promote tumor progression by regulating angiogenesis, enhancing inflammatory response and tumor cell dissemination, and suppressing immune response^8^,^9^,^10^,^11^,^12^,^13^. However TAMs have short lifespan and non-proliferative property, they have to continuously be recruited into tumor^14^,^15^. Therefore after delivery of TAMs as drug carriers into central hypoxic region in the tumor, elimination or destruction of TAMs with tumor hypoxic region, may induce tumor suppression by killing tumor cells and prevent metastasis of tumor cells. Thus, macrophage-mediated drug delivery can provide a strategy to carry therapeutic agents to malignant solid tumors.

A magnetic-based cell therapy, which consists of magnetic particle-loaded cells that can be delivered into target tissue by magnetic target system has recently been proposed as an advanced cell therapy^16^. When disease is generated in multiple sites or in an inaccessible tissue, therapeutic cells only have to be injected systemically^14^. However, magnetic-based cell therapy facilitates the precise delivery of a therapeutic drug with magnetic NPs (MNPs) to a specific target lesion^17^. The magnetic particle-containing cells can be traced or delivered to diverse organs using an external magnetic field in a fluid environment such as blood vessels, lymphatic vessels, or the urinary tract^17^. In addition, magnetic-based drug delivery has particular therapeutic potential for inaccessible organs such as brain and liver. Especially, some types of tumors are in a hypoxic state in the center of an abnormal vascular network, thus lowering the therapeutic effects of radiotherapy or chemotherapy^17^; the therapeutic effects can be improved by using EMA system.

Biodegradable PLGA NPs have been widely used for drug delivery in tumor therapy as carriers of antitumor agents, DNA encoding, and tumor-associated antigens^18^. PLGA, which has been approved for human use by the US Food and Drug Administration, has great potential as a carrier of peptides, DNA, and antigens^19^,^20^,^21^. In addition, PLGA NPs possess many advantages as carriers in a delivery system, such as non-toxicity, easy cell internalization, protection of the loaded agents from degradation, and prolonged drug release^18^. Moreover, internalized PLGA particles with 0.1–10 μm diameters in particular can generate a cytotoxic CD8 + T lymphocyte response by antigen presentation of immune cells^22^, which can elevate tumor-associated immune responses. As such, PLGA particle size influences the stability of intracellular PLGA particles, the amount of drug loading, and the drug release rate^23^.

In this study, we propose an active, hybrid-actuating, macrophage-based microrobot for drug delivery, which can be actuated not only by an EMA system, but also by the tumor infiltration properties of macrophages. The macrophage-based microrobots are fabricated using the phagocytosis of a mouse origin macrophage cell line (J774A.1) to iron oxide (Fe_3_O_4_) NPs and docetaxel (DTX)-encapsulated poly-lactic-co-glycolic acid (PLGA) nanoparticles. An EMA system is used for the macro-manipulation mechanism of the macrophage-based microrobot. Finally, the targeting and therapeutic effects of the proposed macrophage-based microrobots toward different types of tumors are qualitatively and quantitatively evaluated using microfluidic channel, live cell imaging technique, and cytotoxicity tests.

## Results

### Fabrication of macrophage-based microrobots

**Figure 1:**
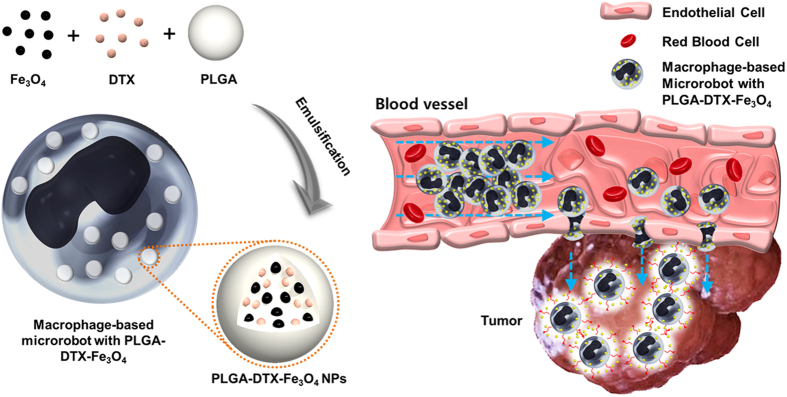
Development of macrophage-based microrobot and therapy. Schematic diagram of the macrophage-based microrobot with PLGA-DTX- Fe_3_O_4_ (left); depiction of possible tumor targeting and therapy in an *in vivo* environment (right).

Figure 1 presents the schematic design concept of macrophage-based microrobots for the active, targeted delivery of anticancer drugs to solid tumors. The macrophage-based microrobots were fabricated using the internalization activities (phagocytosis) of immune cells (macrophages) when the macrophages were co-incubated with PLGA NPs containing Fe_3_O_4_ and an anti-tumor drug^24^,^25^. To induce the therapeutic effects of the macrophage-based microrobots against tumors, DTX was encapsulated in the PLGA NPs. The macrophage-based microrobots were designed to have hybrid actuation through active tumor targeting by the EMA system and infiltration into the tumor tissue by macrophage recruitment. Therefore, first, to guide the macrophage-based microrobots to the sites of interest, such as tumors, Fe_3_O_4_ NPs were loaded into the PLGA NPs. Then, the motions of the macro-based microrobots could be controlled using an external EMA system. Second, using the self-actuating abilities of the macrophage, the macrophage-based microrobots were able to infiltrate into the tumor tissue and release the loaded therapeutic agents to kill the tumor tissue or inhibit its growth (i.e., suicide-bombing microrobots).

**Figure 2:**
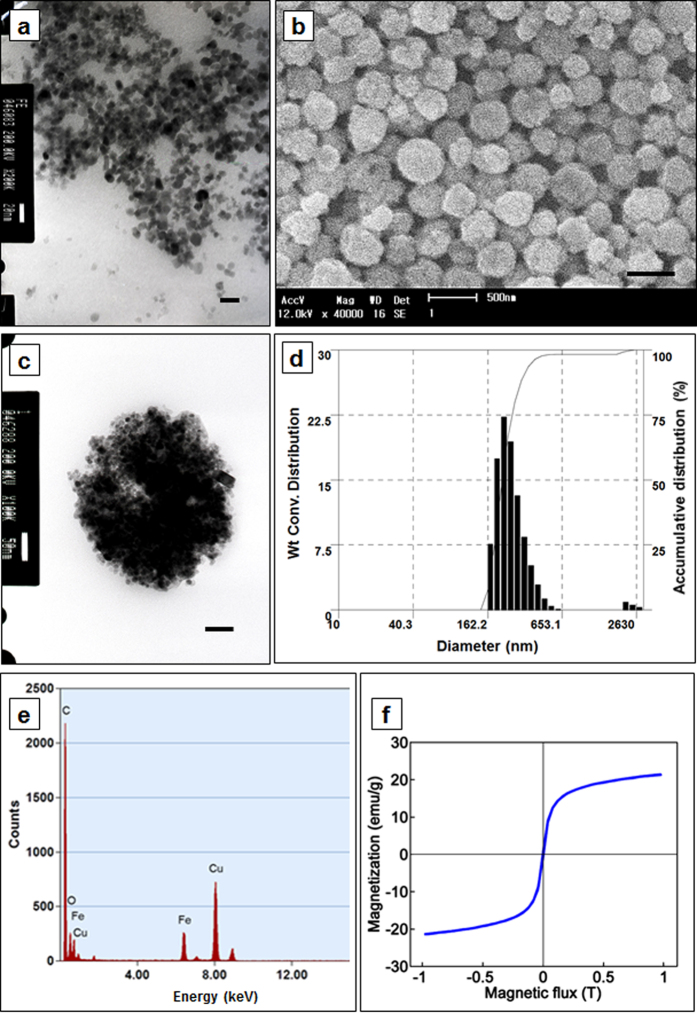
Microscopy images of PLGA-DTX-Fe_3_O_4_ nanoparticles. (**a**) TEM image of MNP nanoparticles, their size was 7–8 nm in diameter. (**b**,**c**) SEM, TEM images of PLGA-DTX- Fe_3_O_4_ NPs, and (**d**) Size distribution of PLGA-DTX-Fe_3_O_4_ NPs; average size, 300 nm in diameter, (**e**) EDX elementary analysis for identification of Fe distribution in PLGA-DTX- Fe_3_O_4_ NPs. (**f**) Magnetization curve of the PLGA-DTX-Fe_3_O_4_ NPs.

In this study, we prepared PLGA NPs containing Fe_3_O_4_ and DTX for cellular internalization. First, biocompatible Fe_3_O_4_ NPs were synthesized using a modified, controlled chemical co-precipitation method to obtain Fe_3_O_4_ NPs that were ultrafine, uniform, and nearly spherical, which could be well dispersed in an aqueous solution due to COO- on the surfaces of the NPs^26^. In this method, a mixture of ferrous/ferric salt solution was used in an alkaline medium, and sodium oleate was used as a surfactant. Figure 2a shows a transmission electron microscope (TEM) image of the fabricated Fe_3_O_4_ NPs with a mean diameter of 10 nm. The PLGA-DTX-Fe_3_O_4_ NPs were prepared using a single emulsion method^14^,^27^. The fabricated PLGA-DTX-Fe_3_O_4_ NPs had spherical surfaces and homogeneous sizes with an average diameter of 300 nm, as shown in a scanning electron microscope (SEM) image (Fig. 2b), TEM image (Fig. 2c) and a size distribution chart (Fig. 2d). The successful encapsulation of the Fe_3_O_4_ NPs into the PLGA-DTX-Fe_3_O_4_ NPs was confirmed by the TEM image and the EDX mapping, which showed the even distribution of the Fe_3_O_4_ NPs (Fig. 2d) and the qualitative inclusion of Fe element inside the PLGA-DTX-Fe_3_O_4_ NPs (Fig. 2e), respectively. In addition, the magnetization of the fabricated PLGA-DTX-Fe_3_O_4_ NPs was measured (Fig. 2f).

**Figure 3:**
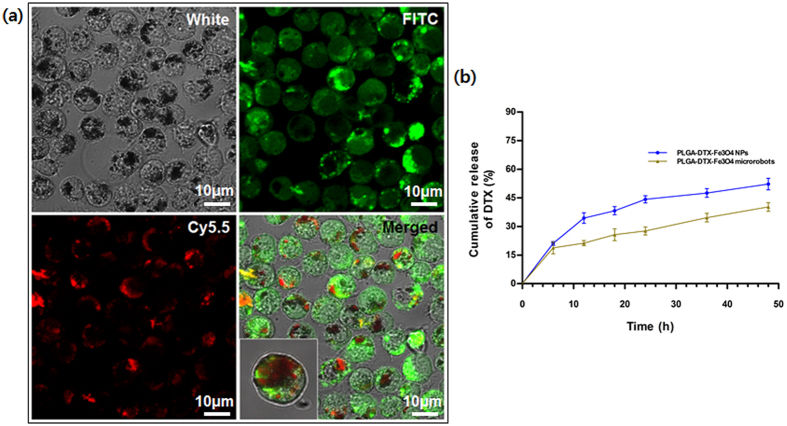
Confirmation of internalization of PLGA-DTX-Fe_3_O_4_ NPs into macrophages. (**a**) Identification of intracellular fluorescence from PLGA-DTX-Fe_3_O_4_ NPs by confocal microscopy. PLGA-DTX-Fe_3_O_4_ NPs were linked with streptavidin-PerCP-Cy5.5 (red) and the macrophages were stained using FITC (green) (Scale bar 10 μm). (**b**) Cumulative release rate of DTX from macrophage-based microrobots with PLGA-DTX-Fe_3_O_4_ and NPs.

The internalization of the PLGA-DTX-Fe_3_O_4_ NPs into the macrophages was accomplished via the phagocytosis of the macrophages, which was induced by co-culturing them^25^. Because macrophages innately carry phagocytosis capacity, diverse therapeutic NPs can be embedded easily into the macrophages^8^. To confirm the engulfment, the carboxylic functional groups of the PLGA-DTX-Fe_3_O_4_ NPs were covalently linked with streptavidin-PerCP-Cy5.5, and the macrophages were stained using fluorescein isothiocyanate (FITC). As shown in Fig. 3a, the signal of Cy5.5 (red) was well detected within the macrophages (green) under a confocal microscope. In addition, the accumulative drug release of DTX from the macrophages after the PLGA-DTX-Fe_3_O_4_ NPs internalization, was measured (Fig. 3b). These results confirmed the successful fabrication of the PLGA-DTX-Fe_3_O_4_-NP-engulfed, macrophage-based microrobots for chemotherapeutic functions.

### Motility evaluation of macrophage-based microrobots in a microfluidic channel

**Figure 4:**
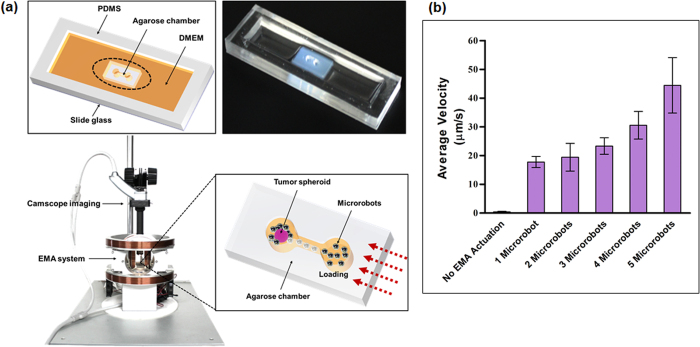
Motility enhancement of macrophage-based microrobots in a channel. (**a**) (top) Microfluidic channel for evaluating tumor-targeting properties of macrophage-based microrobots; (bottom) comprehensive system for external active actuation and imaging of macrophage-based microrobots (bottom). (**b**) Velocities of the microrobots showing different speeds by number of aggregated cells.

It was expected that the macrophage-based microrobots would be able to exhibit advanced tumor-targeting properties, as macrophages possess innate tumor-infiltration characteristics and macrophage-based microrobots with internalized MNPs can be manipulated using an EMA system. To evaluate their motility, we fabricated a microfluidic chamber, which was introduced in our previous report^28^. This microfluidic channel can simply maintain a stable and uniform concentration of chemoattractant with no flow, which enables the confirmation of the tumor-targeting properties of the macrophages and MNPs. This system consists of two chambers—one chamber was filled with tumor cell spheroids or lysates and the other chamber was loaded with the microrobots (Fig. 4a).

**Figure 5:**
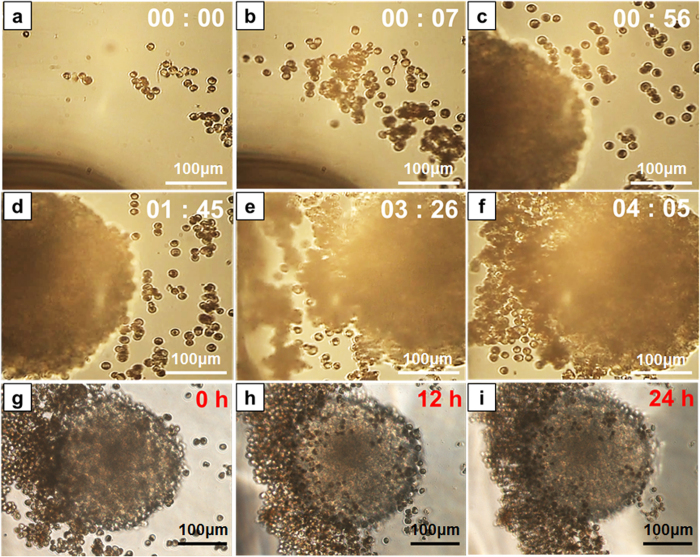
Evaluation of active tumor targeting of macrophage-based microrobots. (**a–f**) Active targeting of microrobots toward tumor spheroid using external magnetic field for 10 min (Scale bar 100 μm). (**g**–**i**) Tumor spheroid attachment property of the microrobots due to the intrinsic tumor infiltration characteristics of the macrophages (Scale bar 100 μm).

At first, the tumor infiltration property of the macrophages was evaluated using tumor spheroids with 4T1 and CT-26 cells, and only macrophages. After co-incubation of the macrophages and the tumor spheroid for 24, the macrophages were observed in and around the tumor spheroids. Therefore, we could confirm that the macrophages were attached on the surfaces of the tumor spheroids or infiltrated around the spheroids (Supplementary Information, Fig. S1). Then, to evaluate the external actuation of the microrobots, the microfluidic channel was placed in the middle of the region of interest (ROI) in the EMA system (Fig. 4a), which generated an actuation force on the MNP-containing, macrophage-based microrobots with a uniform gradient magnetic field of 10 mT/m. The motion of the microrobots was recorded using a CamScope, and their velocity was calculated from the recorded images by a tracking algorithm using MATLAB. The microrobots were loaded into one chamber and the tumor spheroid was loaded into the other chamber. Then, the magnetic field was applied for ten minutes, from the microrobot chamber towards the tumor spheroid chamber. Through the magnetic actuation force of the EMA system, the microrobots were able to move toward the spheroid. Some of the microrobots moved separately, but most of them exhibited collective movement. The microrobots moved at different velocities, ranging 17–63 μm/sec according to the aggregation of the microrobots, as shown in Fig. 4b. When we removed the magnetic actuation force, the movement of the microrobots was significantly reduced at 0.4 μm/sec (Fig. 4b). During a four-minute application of the magnetic field, most of the loaded macrophage-based microrobots attached to the tumor spheroid (Fig. 5f). The magnetic field was then turned off, and the tumor spheroid and infiltrated microrobots were incubated and monitored for 24 h at 37 °C and 5% CO_2_ (Fig. 5g–i). Most of the microrobots that had migrated toward the tumor spheroid showed infiltration in and around the spheroid by the tumor infiltration property of the macrophages during the incubation. The microrobots that attached to the surfaces of the tumor spheroid immediately after the EMA was turned off showed the same properties after 24 h. The microrobots near the spheroid also moved toward it; they subsequently attached to and accumulated on the surface of the tumor spheroid after 24 h (Fig. 5g–i).

### Confirmation of the tumor-killing effects of the macrophage-based microrobots

**Figure 6:**
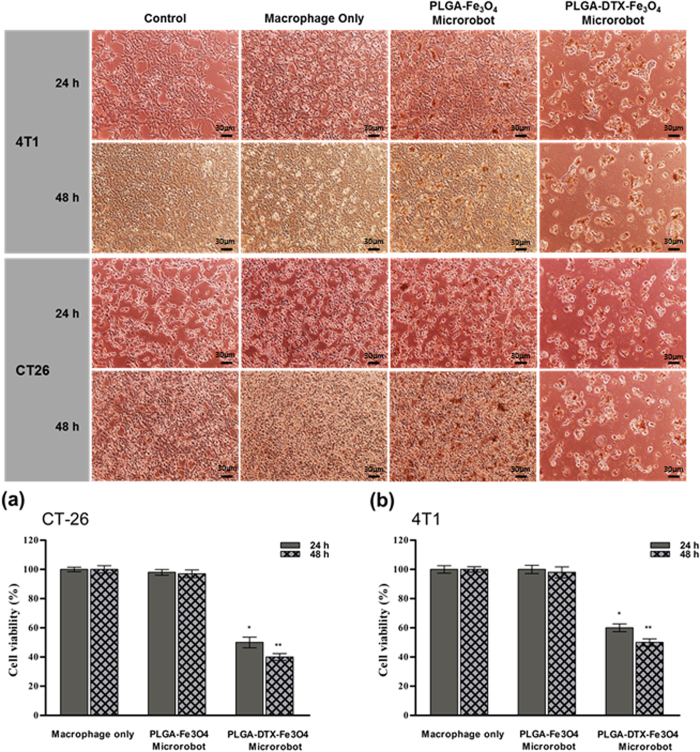
Chemotherapeutic effects of macrophage-based microrobots. (Top) Microscopy images of tumor cell lines treated with macrophage-based microrobots with PLGA-DTX-Fe_3_O_4_ (Scale bar 30 μm). (Bottom) Quantification of cytotoxicity macrophage-based microrobots with PLGA-DTX- Fe_3_O_4_ against cancer cell lines ((**a**) 4T1, (**b**) CT-26).

The cytotoxicity of the proposed macrophage-based microrobots was evaluated qualitatively using an inverted microscope. For this purpose, the morphologies of the 4T1 and CT-26 cells were captured at 24 and 48 h after treatment with different samples: macrophages only, macrophage-based microrobots without drug (macrophage-based microrobots with PLGA-Fe_3_O_4_), and macrophage-based microrobots with drug (macrophage-based microrobot with PLGA-DTX-Fe_3_O_4_). As shown in Fig. 6, the macrophages only and the macrophage-based microrobots without drug had almost no therapeutic effects on the cancer cells. In contrast, the proposed macrophage-based microrobots with the drug induced considerable therapeutic effects against the cancer cells, evidenced by significant changes and decreased cell morphology after 24 and 48 h.

The therapeutic effects of the macrophage-based microrobots were evaluated quantitatively using MTT assay results. As indicated in Fig. 6b, the viability of the cancer cells after treatment with the macrophages only and with the macrophage-based microrobots without the drug remained over 90% after 24 and 48 h. However, when treated with macrophage-based microrobots with a drug concentration of 20 ng/ml, the viability of the 4T1 cells decreased to about 60% after 24 h and 50% after 48 h, and the viability of the CT-26 cells decreased to about 50% after 24 h and 40% after 48 h.

## Discussion

In this study, macrophage-based microrobots were fabricated that consisted of PLGA NPs containing Fe_3_O_4_, the drug DTX, and the macrophage, and their tumor-targeting and therapeutic effects were evaluated. NPs can be internalized efficiently by cells through endocytosis^29^,^30^, but they have exhibited rapid leakage from cells by exocytosis unless they are attached to the cell membrane^31^,^32^. In addition, NPs (<200 nm in diameter) representatively have accommodated lower loaded drug concentrations and displayed earlier drug release rates compared with larger particles, whereas particles ~1 μm in diameter have remained stably within cells for several weeks^23^.

PLGA NPs 300 nm in diameter, containing Fe_3_O_4_ and DTX were fabricated and internalized into macrophages through co-incubation for 12 h. These macrophages modified with PLGA NPs containing Fe_3_O_4_ and an antitumor drug showed no changes in cell phenotypes, including attachment property, viability, and proliferation rate. However, these macrophage-based microrobots displayed active actuation toward a tumor spheroid through EMA (Fig. 5) and demonstrated considerable therapeutic effects against 4T1 and CT-26 tumor cell lines (Fig. 6). Therefore, in this study, macrophages were used as vectors for drug delivery to tumor areas. The drug- and MNP-containing macrophages were actively actuated through the interaction between the MNPs and the EMA system. Moreover, infiltration of the tumor environment was indicated by the innate macrophage characteristics and cytotoxic effects on tumor cells caused by the chemotherapeutic effect of the contained agent.

Magnetic based drug delivery system, which uses magnetically reacting objects, therapeutic agents and magnetic field generators (e.g. permanent magnet or EMA system), has been applied to enhance therapeutic drug delivery to target regions of tumors, infections, ischemia and other diseases^33^,^34^,^35^,^36^. The magnetic field can physically guide the therapeutic drugs to target disease region, which can reduce the side effects of chemotherapy by low drug concentration of other organs. This magnetic based drug delivery system can be also used to enhance the drug targeting through the penetration into cellular barriers, such as brain blood barrier (BBB) and round window membrane (RWM) in inner ear^33^,^37^. In these cases, the magnetic fields generated the pulling force of the drug containing magnetic particles, improved the penetration of the drug into the barriers of brain and inner ear, and enhanced the therapeutic effect. In addition, the magnetic based delivery system can be also used in a cell-based regenerative medicine therapy using stem cells^38^, where magnetic particle labeled cells can be guided to the target region and retained by a magnetic field. Over the past 15 years, magnetic cell therapy has gained interest as a modification for improving the delivery of therapeutic cells to a target region and prolonging their presence there^38^. Preclinical reports have stated that magnetic cell therapy has resulted in 1.5–30-fold greater cell delivery to a target region and a sustained cell population compared non-magnetic cell therapy^38^,^39^,^40^,^41^,^42^,^43^. Consequently, magnetic cell therapy might reduce the costs associated with cell production by requiring fewer cell injections, side effects such as off-targeting due to loss of cells, enhancing therapeutic effects.

In conclusion, we fabricated active actuating, macrophage-based microrobots containing a therapeutic agent for cancer therapy, and we evaluated their targeting performance and therapeutic effects. The macrophages demonstrated efficient internalization of the therapeutic agents and MNPs due to their innate phagocytic characteristics. The macrophage-based microrobots exhibited hybrid actuation of the active electromagnetic actuation toward a tumor spheroid by an EMA system and efficient recruitment around the tumor spheroid due to the intrinsic tumor infiltration property of the macrophages. Finally, the chemotherapeutic effects of the macrophage-based microrobots against tumor cell lines were verified. In the future, in order to apply these active actuating, macrophage-based microrobots to clinical applications in cancer therapy, the fabrication procedures used should be optimized and their performance should be verified through *in vitro* and *in vivo* experiments.

## Methods

### Preparation of antitumor drug-encapsulated magnetic nanoparticles (PLGA-DTX-Fe_3_O_4_ NPs)

First, Fe_3_O_4_ NPs were prepared using a chemical co-precipitation technique^26^. Briefly, 1.622 g (10 mM) iron (III) chloride and 0.634 g (5 mM) iron (II) chloride were dissolved in 12 mL of a hydrochloride aqueous solution (HCl 1 M) to synthesize the Fe_3_O_4_ NPs. Then, this solution was added to 50 mL of hydroxide aqueous solution (NaOH 1 M) containing 3 g of oleic acid, with vigorous stirring for 60 min under protection of dry nitrogen at 60 °C. At this step, the color of the solution turned from light brown to black, and the Fe_3_O_4_ NPs were formed and precipitated. Next, the precipitates were washed by repeated cycles of centrifugation and re-dispersed into an absolute alcohol solution. The washing process was performed three times, after which the final Fe_3_O_4_ NPs were dried in a vacuum chamber at room temperature for 48 h. The PLGA-DTX-Fe_3_O_4_ NPs were prepared using a single emulsion method^27^. Briefly, the PLGA (Sigma-Aldrich Chemical, St. Louis, MO), Fe_3_O_4_ NPs, and DTX (Samyang Biopharmaceutical Corp., Daejeon, Korea), in a mass ratio of 10:10:1, were dissolved in 2 mL dichloromethane and mixed vigorously for 15 min (oil phase). The solution was then poured into 40 mL PVA 1% (water phase) to make an oil-in-water system. The droplets of PLGA-DTX-Fe_3_O_4_ NPs in the oil-in-water system were emulsified by sonication (VC750; Sonics & Materials, Newton, CT), and the solution, including the fabricated droplets, was placed in a fume hood overnight to evaporate the solvent. Then, the PLGA-DTX-Fe_3_O_4_ NPs were washed five times with deionized water (DI water) and stored at 4 °C. The sizes of the PLGA-DTX-Fe_3_O_4_ NPs were evaluated using a particle size analyzer (ELS-8000; Otsuka Electronics, Osaka, Japan), and the surface morphologies of the PLGA-DTX-Fe_3_O_4_ NPs were investigated using a SEM (SS-550; Shimadzu, Kyoto, Japan). A TEM and a confocal laser scanning microscope (TCS SP5/AOBS Tandem; Leica, Wetzlar, Germany) were used to confirm the encapsulation of the DTX and Fe_3_O_4_ NPs in the PLGA-DTX-Fe_3_O_4_ NPs.

### Cell culture

A mouse macrophage cell line, J774A.1, was obtained from Sigma-Aldrich. The mammary carcinoma cell line 4T1 and the murine colorectal cancer cell line CT-26 were purchased from American Type Culture Collection (Manassas, VA). The J774A.1 cells were maintained in RPMI 1640 supplemented with 10% fetal bovine serum (FBS), penicillin, and streptomycin, purchased from Gibco BRL (Gaithersburg, MD). The 4T1 and CT-26 cells were cultured in Dulbecco’s Modified Eagle’s medium (DMEM) (Lonza, Walkersville, MD) containing 10% FBS and 1% (v/v) antibiotics. The J774A.1 cells were collected using a cell scraper and the other cells were dissociated using 0.5% trypsin-EDTA (Life Technologies, Grand Island, NY). All cells were centrifuged at 1200 rpm for 3 min, and the obtained pellets were suspended in the growth medium.

### Motility evaluation of macrophage-based microrobot in a channel

Figure 3a depicts the experimental setup for evaluating the motility of the macrophage-based microrobots. The experimental setup consisted of the microfluidic channel, the EMA system, and a CamScope imaging apparatus (CamScope PLUS; Sometech, Seoul, Korea).

### Fabrication of motility evaluation channel

The motility chamber was designed to test the controllability of the microrobots carrying Fe_3_O_4_ NPs and DTX-encapsulated PLGA NPs using an EMA system. As shown in Fig. 3a, the chamber consisted of a polydimethylsiloxane (PDMS) frame attached to a slide glass, an agarose chamber containing two working chambers connected by microtubing, with DMEM solution outside the agarose chamber. One of the two working chambers was used to load the microrobots and the other one was used to load the tumor spheroid. Using the EMA system, the macrophage-based microrobots could be controlled to target the tumor spheroid and inhibit its growth by releasing the encapsulated drug. The motility chamber was fabricated using conventional micro-molding of hydrogel patterns. Briefly, a mold with a dumbbell-like structure was fabricated in a 3-D printer (Objet30 Pro; Stratasys, Eden Prairie, Minnesota, USA) and a rectangular PDMS frame was prepared and attached to the slide glass using an O_2_ plasma asher. Then, 2% (w/v) agarose in DI water was poured into the PDMS frame, covering the mold, which was placed in the center of the chamber. After the agarose channel had cured at room temperature, the mold was removed to make the working chambers. To maintain enough medium for the experiments, DMEM was added to the section between the PDMS frame to diffuse into the working chambers through agarose, as shown in Fig. 3a.

### Electromagnetic actuation (EMA) system

An EMA system was adopted for the external active actuation of the macrophage-based microrobot, as shown in Fig. 3a. The EMA system consisted of three pairs of Helmholtz coils-one pair each on the x-axis, y-axis, and z-axis—and two pairs of Maxwell coils—one pair each on the x-axis and y-axis. The role of the three pairs of Helmholtz coils was to create a uniform magnetic flux, while the purpose of the two pairs of Maxwell coils was to induce a uniform gradient of magnetic flux in the ROI. The motion of the microrobots in the ROI of the EMA system could be controlled by the currents of the EMA system using an NI PXI controller and the LabVIEW program (National Instruments, Austin, TX). The induced torque (**T**) and propulsive force (**F**) on the microrobot could be determined as


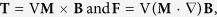


where V, **M,** and **B** are the volume and magnetization of the microrobot and the magnetic flux in the ROI of the EMA system, respectively; ∇ denotes the gradient symbol^44^,^45^.

### Preparation of tumor spheroids

The 4T1 and CT-26 tumor spheroids were prepared^16^. Briefly, a confluent culture of the cells was harvested using 0.5% trypsin-EDTA, washed in PBS, centrifuged, and suspended in DMEM. After that, 20 μL drops of the cell suspension containing 10^4^ cells were placed on the inner surfaces of the covers of 10 cm cell culture dishes prepared with 10 mL DMEM. The hanging drops of cell cultures over the medium were incubated in a humidified incubator. After enough growing time elapsed (about six days), the cellular tumor spheroids were obtained and harvested using a pipette.

### Qualitative tumor-killing effects of macrophage-based microrobots by live-cell imaging

The migration and attachment of the macrophage-based microrobots toward the tumor spheroids were evaluated using live-cell imaging (CU-109; Live Cell Instrument, Seoul, Korea) and observed under an inverted microscope (Ti-U; Nikon USA, Melville, NY). Specifically, the harvested 4T1 and CT-26 tumor spheroids were prepared in 10 mL cell culture dishes containing DMEM. The spheroids were then treated with macrophage-based microrobots with PLGA-DTX-Fe_3_O_4_. For comparison, the spheroids were also treated with macrophages only and with macrophage-based microrobots without the drug (macrophage-based microrobots with PLGA-Fe_3_O_4_). Finally, the morphology changes of the spheroids were measured using the live-cell imaging apparatus and the inverted microscope.

### *In vitro* drug release study

The *in vitro* drug release of DTX from the PLGA-DTX-Fe_3_O_4_ NPs and the macrophage-based microrobots with PLGA-DTX-Fe_3_O_4_ were examined using the dialysis bag technique^46^,^47^. Briefly, 100 μg of DTX in PLGA-DTX-Fe_3_O_4_ NPs and the same volume of the drug in the macrophage-based microrobots were prepared in dialysis bags with molecular weight cutoff of 3500 Da, which were immersed in 50 mL tubes containing 20 mL PBS (pH = 7) with 0.1% (w/v) Tween 80 for sink conditions. The release conditions were maintained by keeping the tubes in a shaking incubator at 37 °C and 100 rpm. At each predetermined time interval, 1 mL of the releasing medium was withdrawn and the same volume of PBS was added to the tubes. The drug content in each sample was determined using high-performance liquid chromatography (Shimadzu, Kyoto, Japan). Based on the obtained data, the drug release profiles of DTX in the PLGA-DTX-Fe_3_O_4_ NPs and the macrophage-based microrobots with PLGA-DTX-Fe_3_O_4_ were calculated.

### Cytotoxicity test of the macrophage-based microrobots on 4T1 cells

The cytotoxicity of the macrophage-based microrobots was investigated using MTT assays on the 4T1 and CT-26 cell lines. First, the cells were cultured in 96-well plates (SPL life Sciences, Gyeonggi, Korea) at a density of 10^4^ cells/well in 100 μL of the culture medium and incubated overnight in an incubator at 37 °C and 5% CO_2_ for cell attachment. The used medium was removed and the cells were incubated with different samples: macrophages only (10^4^ cells/well); macrophage-based microrobots without the drug (macrophage-based microrobots with PLGA-Fe_3_O_4_, 10^4^ cells/well); and macrophage-based microrobots with the drug (macrophage-based microrobots with PLGA-DTX-Fe_3_O_4_, 10^4^ cells/well) in 100 μL culture medium. The drug concentration in the drug-containing samples was adjusted to 20 ng/mL. After 24 h or 48 h, the spent medium was discarded and the wells were washed twice with PBS (pH = 7). Next, the cells in each well were prepared with MTT (Sigma-Aldrich) in the culture medium at a concentration of 0.5 mg/mL and further incubated for 3.5 h. The medium was replaced with 100 μL of dimethyl sulfoxide (Duksan Pure Chemicals, Gyeonggi, Korea). Finally, the viability of the cells was measured using a micro plate reader (Thermo Scientific, Waltham, CA) at a wavelength of 570 nm.

### Statistical Analysis

The data were shown as means with standard deviations of three samples. Comparisons of the data were performed using Student’s t test; *represents *P* < 0.05 and **represents *P* < 0.01. All experiments were repeated at least three times on separate days, and the data shown in this paper are representative of all of the repetitions.

## Additional Information

**How to cite this article**: Han, J. *et al*. Hybrid-Actuating Macrophage-Based Microrobots for Active Cancer Therapy. *Sci. Rep.* **6**, 28717; doi: 10.1038/srep28717 (2016).

## Supplementary Material

Supplementary Information

## References

[b1] Kobayashi H., Watanabe R. & Choyke P. L. Improving conventional enhanced permeability and retention (EPR) effects; what is the appropriate target? Theranostics 4, 81–89 (2014). 24396516 10.7150/thno.7193 PMC3881228

[b2] Alexis F., Pridgen E., Molnar L. K. & Farokhzad O. C. Factors affecting the clearance and biodistribution of polymeric nanoparticles. Mol. Pharm. 5, 505–515 (2008). 18672949 10.1021/mp800051m PMC2663893

[b3] Fang J., Nakamura H. & Maeda H. The EPR effect: Unique features of tumor blood vessels for drug delivery, factors involved, and limitations and augmentation of the effect. Adv. Drug. Deliv. Rev. 18, 136–151 (2011). 20441782 10.1016/j.addr.2010.04.009

[b4] Maki S., Konno & Maeda T. H. Image enhancement in computerized tomography for sensitive diagnosis of liver cancer and semiquantitation of tumor selective drug targeting with oily contrast medium. Cancer 56, 751–757 (1985). 3160453 10.1002/1097-0142(19850815)56:4<751::aid-cncr2820560409>3.0.co;2-y

[b5] Nagamitsu A., Greish K. & Maeda H. Elevating blood pressure as a strategy to increase tumor-targeted delivery of macromolecular drug SMANCS: cases of advanced solid tumors. Jpn. J. Oncol. 39, 756–766 (2009). 10.1093/jjco/hyp074 19596662

[b6] Madsen S. J., Baek S. K., Makkouk A. R., Krasieva T. & Hirschberg H. Macrophages as cell-based delivery systems for nanoshells in photothermal therapy. Ann. Biomed. Eng. 40, 507–515 (2012). 21979168 10.1007/s10439-011-0415-1 PMC3491981

[b7] Ross J. A. & Auger M. J. The biology of the macrophage. In: Burke B., Lewis C. E. (Eds), The Macrophage, 2nd edition. Oxford University Press, Oxford, 1–72 (2002).

[b8] Choi M. R. . A cellular Trojan Horse for delivery of therapeutic nanoparticles into tumors. Nano. Lett. 7, 3759–3765 (2007). 17979310 10.1021/nl072209h

[b9] Kelly P. M. A., Davison R. S., Bliss E. & McGee J. O. Macrophages in human breast disease: a quantitative immunohistochemical study. Br. J. Cancer 57, 174–177 (1988). 2833921 10.1038/bjc.1988.36 PMC2246436

[b10] Frantz S. & Nahrendorf M. Cardiac macrophages and their role in ischaemic heart disease. Cardiovasc. Res. 102, 240–248 (2014). 24501331 10.1093/cvr/cvu025 PMC3989449

[b11] Tripathi C. . Macrophages are recruited to hypoxic tumor areas and acquire a pro-angiogenic M2-polarized phenotype via hypoxic cancer cell derived cytokines Oncostatin M and Eotaxin. Oncotarget. 30, 5350–6538 (2014). 25051364 10.18632/oncotarget.2110 PMC4170629

[b12] Zhang B. C. . Tumor-associated macrophages infiltration is associated with peritumoral lymphangiogenesis and poor prognosis in lung adenocarcinoma. Med. Oncol. 28, 1447–1452 (2011). 20676804 10.1007/s12032-010-9638-5

[b13] Negus R. P. M., Stamp G. W. H., Hadley J. & Balkwill F. R. Quantitative assessment of the leukocyte infiltrate in ovarian cancer and its relationship to the expression of C-C chemokines. Am. J. Pathol. 150, 1723–1734 (1997). 9137096 PMC1858213

[b14] Movahedi K. . Different tumor microenvironments contain functionally distinct subsets of macrophages derived from Ly6C (high) monocytes. Cancer Res. 70, 5728–5739 (2010). 20570887 10.1158/0008-5472.CAN-09-4672

[b15] Cortez-Retamozo V. . Origins of tumor-associated macrophages and neutrophils. Proc. Natl.Acad. Sci. USA 109, 2491–2496 (2012). 22308361 10.1073/pnas.1113744109 PMC3289379

[b16] Muthana M. . Directing cell therapy to anatomic target sites *in vivo* with magnetic resonance targeting. Nat. Commun. doi: 10.1038/ncomms9009 (2015). PMC4568295 26284300

[b17] Shen S. . Preliminary design towards a magnetic actuated drug delivery system. IEEE 7th International conference on CIS&RAM, pp. 245–249 (2015).

[b18] Krishnamachari Y., Geary S. M., Lemke C. D. & Salem A. K. Nanoparticle delivery systems in cancer vaccines. Pharm. Res. 28, 215–236 (2011). 20721603 10.1007/s11095-010-0241-4 PMC3559243

[b19] Gupta R. K., Chang A. C. & Siber G. R. Biodegradable polymer microspheres as vaccine adjuvants and delivery systems. Dev. Biol. Stand. 92, 63–78 (1998). 9554260

[b20] Hedley M. L., Curley J. & Urban R. Microspheres containing plasmid-encoded antigens elicit cytotoxic T-cell responses. Nat. Med. 4, 365–368 (1998). 9500615 10.1038/nm0398-365

[b21] Helson R. . Polylactide-co-glycolide (PLG) microparticles modify the immune response to DNA vaccination. Vaccine 26, 753–761 (2008). 18191308 10.1016/j.vaccine.2007.12.006

[b22] Kovacsovics-Bankowski M. & Rock K. L. A phagosome-to-cytosol pathway for exogenous antigens presented on MHC class I molecules. Science 267, 243–246 (1995). 7809629 10.1126/science.7809629

[b23] Ankrum J. A. . Engineering cells with intracellular agent-loaded microparticles to control cell phenotype. Nat. Protoc. 9, 233–245 (2014). 24407352 10.1038/nprot.2014.002 PMC4320648

[b24] Thorek D. L. & Tsourkas A. Size, charge and concentration dependent uptake of iron oxide particles by non-phagocytic cells. Biomaterials 29, 3583–3590 (2008). 18533252 10.1016/j.biomaterials.2008.05.015 PMC2518173

[b25] Park S. J. . Monocyte-based microrobot with chemotactic motility for tumor theragnosis. Biotechnol. Bioeng. 111, 2132–2138 (2014). 24771225 10.1002/bit.25270

[b26] Sun J. . Synthesis and characterization of biocompatible Fe_3_O_4_ nanoparticles. J. Biomed. Mater. Res. A. 80, 333–341 (2007). 17001648 10.1002/jbm.a.30909

[b27] Keum C. G. . Practical preparation procedures for docetaxel-loaded nanoparticles using polylactic acid-co-glycolic acid. Int. J. Nanomedicine 6, 2225–2234 (2011). 22114486 10.2147/IJN.S24547 PMC3215163

[b28] Park S. J. . New paradigm for tumor theranostic methodology using bacteria-based microrobot. Sci. Rep. 3, 3394–3401 (2013). 24292152 10.1038/srep03394 PMC3844944

[b29] Panyam J. & Labhasetwar V. Biodegradable nanoparticles for drug and gene delivery to cells and tissue. Adv. Drug Deliv. Rev. 55, 329–347 (2003). 12628320 10.1016/s0169-409x(02)00228-4

[b30] Panyam J., Zhou W.-Z., Prabha S., Sahoo S. K. & Labhasetwar V. Rapid endo-lysosomal escape of poly (DL-lactide-co-glycolide) nanoparticles: implications for drug and gene delivery. FASEB J. 16, 1217–1226 (2002). 12153989 10.1096/fj.02-0088com

[b31] Patil Y., Sadhukha T., Ma L. & Panyam J. Nanoparticle-mediated simultaneous and targeted delivery of paclitaxel and tariquidar overcomes tumor drug resistance. J. Control Release 136, 21–29 (2009). 19331851 10.1016/j.jconrel.2009.01.021

[b32] Acharya S. & Sahoo S. K. PLGA nanoparticles containing various anticancer agents and tumour delivery by EPR effect. Adv. Drug Deliv. Rev. 63, 170–183 (2011). 20965219 10.1016/j.addr.2010.10.008

[b33] Chertok B. . Iron Oxide Nanoparticles as a Drug Delivery Vehicle for MRI Monitored Magnetic Targeting of Brain Tumors. Biomaterials 29, 487–496 (2008). 17964647 10.1016/j.biomaterials.2007.08.050 PMC2761681

[b34] Vandergriff A. C. . Magnetic targeting of cardiosphere-derived stem cells with ferumoxytol nanoparticles for treating rats with myocardial infarction. Biomaterials 35, 8528–8539 (2014). 25043570 10.1016/j.biomaterials.2014.06.031

[b35] Kodama A. . *In vivo* bioluminescence imaging of transplanted bone marrow mesenchymal stromal cells using a magnetic delivery system in a rat fracture model. J. Bone Joint Surg. Br. 94, 998–1006 (2012). 22733960 10.1302/0301-620X.94B7.28521

[b36] Vanecek V. . Highly efficient magnetic targeting of mesenchymal stem cells in spinal cord injury. Int. J. Nanomedicine 7, 3719–3730 (2012). 22888231 10.2147/IJN.S32824 PMC3414205

[b37] Dormer K. J. . Magnetically-targeted, technetium 99 m-labeled nanoparticles to the inner ear. J. Biomed. Nanotech. 4, 1–11 (2008).

[b38] Connell J. J., Patrick P. S., Yu Y., Lythgoe M. F. & Kalber T. L. Advanced cell therapies: targeting, tracking and actuation of cells with magnetic particles. Regen. Med. 10, 757–772 (2015). 26390317 10.2217/rme.15.36

[b39] Consigny P. M., Silverberg D. A. & Vitali N. J. Use of endothelial cells containing superparamagnetic microspheres to improve endothelial cell delivery to arterial surfaces after angioplasty. J. Vasc. Interv. Radiol. 10, 155–163 (1999). 10082102 10.1016/s1051-0443(99)70458-6

[b40] Arbab A. S. . *In vivo* trafficking and targeted delivery of magnetically labeled stem cells. Hum. Gene Ther. 15, 351–360 (2004). 15053860 10.1089/104303404322959506

[b41] Riegler. J. . Targeted magnetic delivery and tracking of cells using a magnetic resonance imaging system. Biomaterials 31, 5366–5371 (2010). 20382425 10.1016/j.biomaterials.2010.03.032

[b42] Riegler J. . Superparamagnetic iron oxide nanoparticle targeting of MSCs in vascular injury. Biomaterials 34, 1987–1994 (2013). 23237516 10.1016/j.biomaterials.2012.11.040

[b43] Riegler J., Allain B., Cook R. J., Lythgoe M. F. & Pankhurst Q. A. Magnetically assisted delivery of cells using a magnetic resonance imaging system. J. Phys. D Appl. Phys. 44, 055001 (2011).

[b44] Choi H., Choi J., Jang G., Park J. O. & Park S. Two-dimensional actuation of a microrobot with a stationary two-pair coil system. Smart Mater. Struct. 18, 055007 (2009).

[b45] Choi H. . Two-dimensional locomotion of a microrobot with a novel stationary electromagnetic actuation system. Smart Mater. Struct. 18, 115017 (2009).

[b46] Muthu M. S., Kulkarni S. A., Raju A. & Feng S. S. Theranostic liposomes of TPGS coating for targeted co-delivery of docetaxel and quantum dots. Biomaterials 33, 3494–3501 (2012). 22306020 10.1016/j.biomaterials.2012.01.036

[b47] Muthu M. S., Kulkarni S. A., J. Xiong J. & Feng S. S. Vitamin E TPGS coated liposomes enhanced cellular uptake and cytotoxicity of docetaxel in brain cancer cells. Int. J. Pharm. 421, 332–340 (2011). 22001537 10.1016/j.ijpharm.2011.09.045

