# Supplementary material for: Hybrid-Actuating Macrophage-Based Microrobots for Active Cancer Therapy
Source: Sci Rep. 2016 Jun 27;6:28717. doi: 10.1038/srep28717 (PMC4921872; doi:10.1038/srep28717)
Supplement: Supplementary Information [file srep28717-s1.pdf]

Hybrid-Actuating Macrophage-Based Microrobots for Active Cancer Therapy

Jiwon Han[1], Jin Zhen[1], Van Du Nguyen[1], Gwangjun Go[1], Youngjin Choi[1], Seong Young Ko[1], Jong-Oh Park[1*], and Sukho Park[1*]

[1]School of Mechanical Engineering, Chonnam National University

Correspondence and requests for materials should be addressed to J. -O. P. or S. P.

Jong-Oh Park, PhD

Mailing address: School of Mechanical Engineering, Chonnam National University, 300, Yongbong-dong, Buk-gu, Gwangju, 500-757, Republic of Korea.

Phone: 82-62-530-1686   Fax: 82-62-530-0267

E-mail: jop@jnu.ac.kr

Sukho Park, PhD

Mailing address: School of Mechanical Engineering, Chonnam National University, 300, Yongbong-dong, Buk-gu, Gwangju, 500-757, Republic of Korea.

Phone: 82-62-530-1687   Fax: 82-62-530-0267

E-mail: spark@jnu.ac.kr

Supplementary Information (Tumor infiltration property of the macrophages)

The tumor infiltration property of the macrophages was evaluated using tumor spheroids with 4T1 and CT-26 cells, and only macrophages. After co-incubation of the macrophages and the tumor spheroid for 24h, the macrophages were observed in and around the tumor spheroids (Figure S1). Therefore, we could confirm that the macrophages were attached on the surfaces of the tumor spheroids or infiltrated around the spheroids.

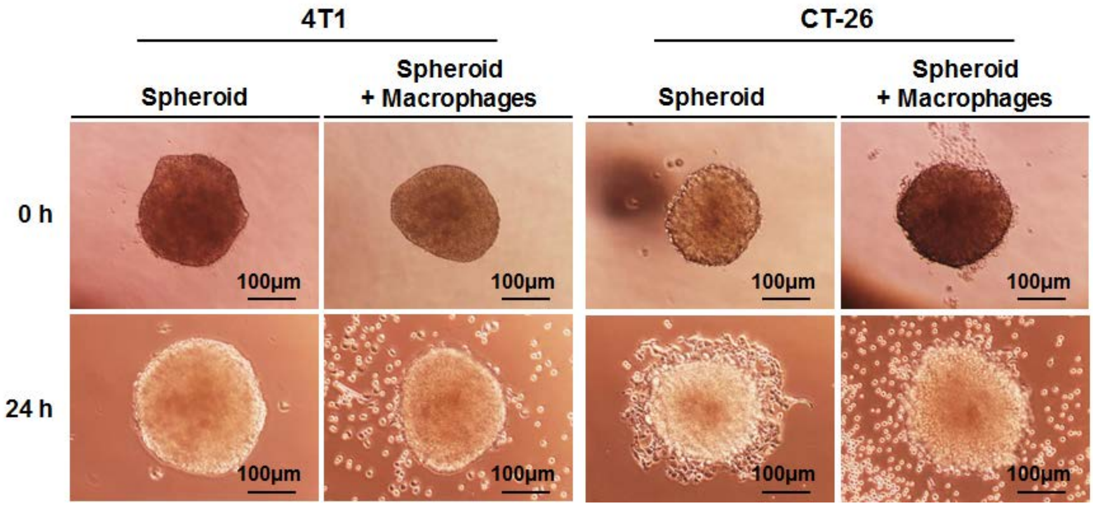

Figure S1 Tumor infiltration property of macrophages. Microscopy images of tumor spheroids (4T1, CT-26) and macrophages after co-culture for 24h (Scale bar 100 μm).
